# ErbB polymorphisms: insights and implications for response to targeted cancer therapeutics

**DOI:** 10.3389/fgene.2015.00017

**Published:** 2015-02-04

**Authors:** Moulay A. Alaoui-Jamali, Grégoire B. Morand, Sabrina Daniela da Silva

**Affiliations:** ^1^Departments of Medicine and Oncology, Segal Cancer Centre and Lady Davis Institute for Medical Research, Sir Mortimer B. Davis-Jewish General Hospital, McGill UniversityMontreal, QC, Canada; ^2^Department of Otolaryngology-Head and Neck Surgery, Sir Mortimer B. Davis-Jewish General Hospital, McGill UniversityMontreal, QC, Canada

**Keywords:** ErbB receptors, cancer, SNPs, anti-ErbB therapeutics, drug response, resistance

## Abstract

Advances in high-throughput genomic-scanning have expanded the repertory of genetic variations in DNA sequences encoding ErbB tyrosine kinase receptors in humans, including single nucleotide polymorphisms (SNPs), polymorphic repetitive elements, microsatellite variations, small-scale insertions and deletions. The ErbB family members: EGFR, ErbB2, ErbB3, and ErbB4 receptors are established as drivers of many aspects of tumor initiation and progression to metastasis. This knowledge has provided rationales for the development of an arsenal of anti-ErbB therapeutics, ranging from small molecule kinase inhibitors to monoclonal antibodies. Anti-ErbB agents are becoming the cornerstone therapeutics for the management of cancers that overexpress hyperactive variants of ErbB receptors, in particular ErbB2-positive breast cancer and non-small cell lung carcinomas. However, their clinical benefit has been limited to a subset of patients due to a wide heterogeneity in drug response despite the expression of the ErbB targets, attributed to intrinsic (primary) and to acquired (secondary) resistance. Somatic mutations in ErbB tyrosine kinase domains have been extensively investigated in preclinical and clinical setting as determinants for either high sensitivity or resistance to anti-ErbB therapeutics. In contrast, only scant information is available on the impact of SNPs, which are widespread in genes encoding ErbB receptors, on receptor structure and activity, and their predictive values for drug susceptibility. This review aims to briefly update polymorphic variations in genes encoding ErbB receptors based on recent advances in deep sequencing technologies, and to address challenging issues for a better understanding of the functional impact of single *versus* combined SNPs in ErbB genes to receptor topology, receptor-drug interaction, and drug susceptibility. The potential of exploiting SNPs in the era of stratified targeted therapeutics is discussed.

## An overview of ErbB receptor signaling

**Figure 1:**
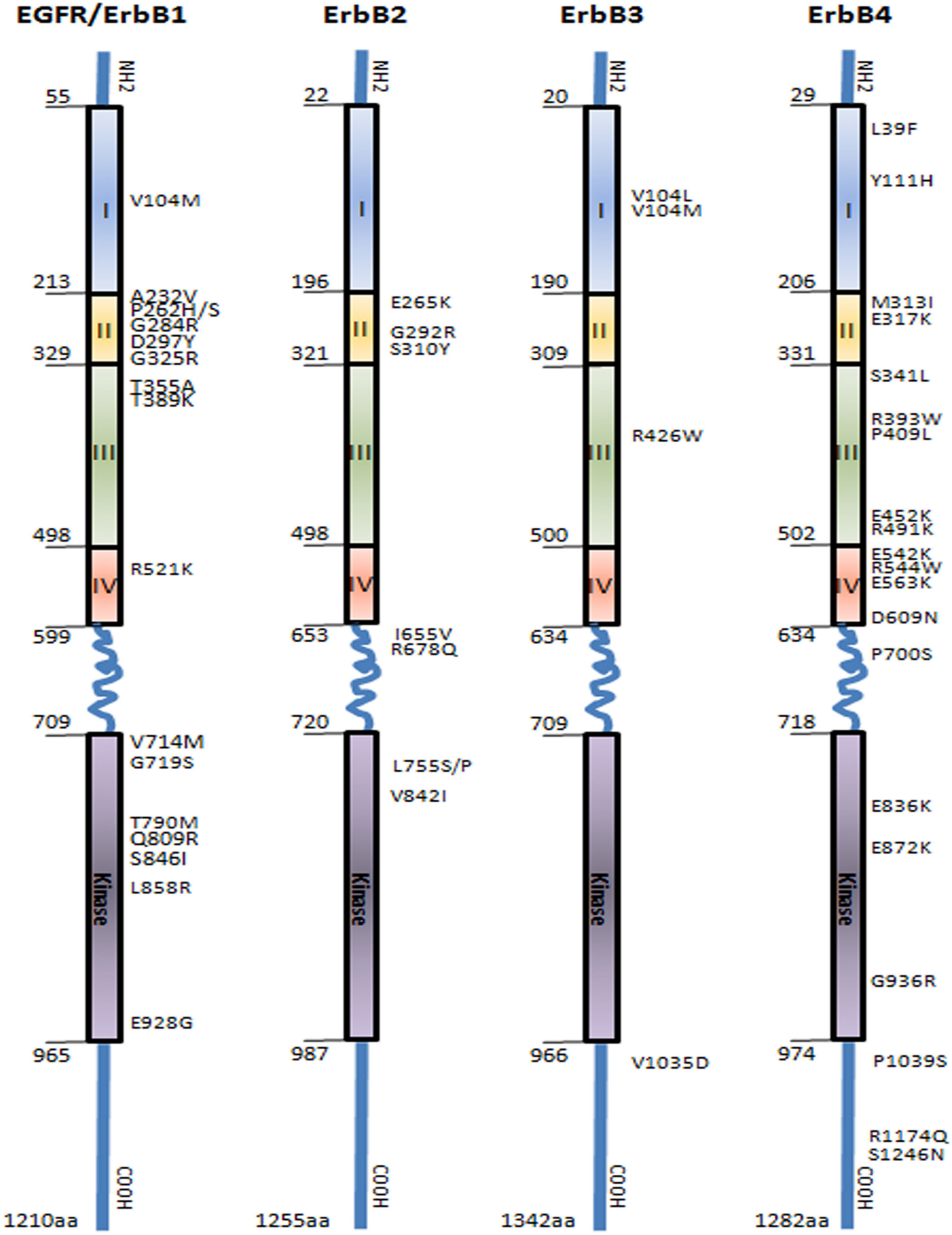
**Representation of epidermal growth factor receptors family (EGFR/ErbB1, ErbB2, ErbB3, and ErbB4) showing the distribution of the main SNPs mutations in the extracellular domain, transmembrane region, and intracellular domain comprising the tyrosine kinase and autophosphorylation sites**. Boxes represent functional domains: I, extracellular domain sub-region I; II, extracellular domain sub-region II; III, extracellular domain sub-region III; IV, extracellular domain sub-region IV. (Prickett et al., 2009; Yarden and Pines, 2012).

Since the pioneering discovery of epidermal growth factor receptor (EGFR), the founding member of the ErbB protein tyrosine kinase family by Stanley Cohen, a contribution recognized as 1986 Nobel Prize of Medicine, a remarkable progress has been made in the characterization of this receptor family, which in addition to EGFR/ErbB1/HER1, includes ErbB2/HER2, ErbB3/HER3, and ErbB4/HER4 (Chinkers and Cohen, 1981). These homologous receptors are type 1 transmembrane proteins that share a common structure with a large glycosylated ligand binding extracellular region composed of domains I-IV, a single hydrophobic transmembrane region, a cytoplasmic domain with tyrosine kinase activity, and a C-terminal tail containing multiple phosphorylation sites required for propagation of downstream signaling (Figure 1). The strongest homology among ErbB receptors is seen in the kinase domain (59–82%) followed by the extracellular domain (36–48%) and the C-terminal tail (24–33%) (Gschwind et al., 2004). There are unique features characterizing two members of this family: the first is the lack of kinase activity in ErbB3 receptor, a deficiency proposed to be due to substitution of a conserved aspartate (Asp/D) residue to an asparagine (Asn/N) in the kinase domain. Therefore, ErbB3 activation relies on heterodimerization with the other members of the family (Citri et al., 2003). The second is the orphan ErbB2, which lacks a specific ligand. Structural studies have revealed that the ligand-binding site of ErbB2 is constitutionally in activate conformation, and thus prone for heterodimerization and transphosphorylation through interactions with the other ligand-activated ErbB receptors (Garrett et al., 2003).

ErbB receptors have a propensity to be activated by a wide range of ligands, which include epidermal growth factor (EGF), transforming growth factor α (TGFα), heparin-binding epidermal growth factor, amphiregulin, betacellulin, epiregulin, and neuregulins. These ligands induce a broad array of ErbB receptor homo- and heterodimerization culminating into a diverse cell transduction signaling of utmost importance to both physiological and pathological contexts, including embryonic development (Gassmann et al., 1995; O-Charoenrat et al., 2002; Stein and Staros, 2006; Birchmeier, 2009). The general mode of activation of ErbB receptors involves changes in allosteric conformations due to intermolecular mechanisms triggered by ligand activation and/or activating mutations (Monsey et al., 2010; Lemmon et al., 2014). Both homo- and heterodimerization with partners involve primarily the kinase domain and C-lobe surface structures of ErbB receptors (Burgess et al., 2003; Zhang et al., 2006; Monsey et al., 2010). For example, the ligand binds between domains I and III (extracellular region) of the EGFR and ErbB3 receptors to induce conformational change and subsequent dimerization and kinase activation. A widely accepted model of activation stipulates that dimerization occurs via an asymmetric mode where a C-lobe of an acceptor monomer and the N-lobe of a donor monomer in the intracellular juxtamembrane region interacts. Mutations in the asymmetric dimer interface can abolish kinase activation (Zhang et al., 2006, 2007; Red Brewer et al., 2009). In this mode of activation, C-lobe surface amino acids, the extracellular domain, the transmembrane domain, and the intracellular juxtamembrane region all can influence receptor dimerization and the intensity of activation (Burgess et al., 2003; Thiel and Carpenter, 2007; Lemmon, 2009). This suggests that subtle molecular changes in the receptor structure due to single nucleotide polymorphisms (SNPs) can impact on ErbB kinase activity as well as ErbB receptor interaction with ligands, downstream partners, or with inhibitors including anti-ErbB monoclonal antibodies (Shigematsu et al., 2005; Fan et al., 2008).

## ErbB receptors: a success story in discovery of therapeutic targets and limitations

In general, ErbB receptors are expressed at low levels on the surface of normal adult epithelial cells (O-Charoenrat et al., 2002). Extensive preclinical studies in cell lines and transgenic mouse models established the contribution of dysfunctional ErbB signaling, due to overexpression and hyperactivation, to carcinogenesis (Bargmann et al., 1986; Bouchard et al., 1989). In cancer cells, aberrant activation due to gene overexpression and/or amplification and (less commonly) mutation scan promote enhanced cell proliferation, resistance to apoptosis, and higher cell invasiveness. In several cancer types, in particular breast, ovarian, gastric, and non-small cell carcinoma of the lung, amplification and/or overexpression of specific members of the ErbB receptor family have been associated with poor clinical prognosis, including high incidence of metastasis and recurrence. In breast cancer, molecular profiling studies have enabled identification of ErbB2-positivebreast cancer molecular subtype, which represents up to 30% of breast cancers. In this subtype, the amplified and/or overexpressed ErbB2 induces enrichment of several downstream ErbB2-regulated genes (Slamon et al., 1989; Cancer Genome Atlas Network, 2012).

The remarkable progress in the understanding the biology of ErbB signaling in cancer has led to the discovery of several targeted agents against ErbB members, including small molecule tyrosine kinase inhibitors (TKIs) and monoclonal antibodies (MAbs). At present, small molecule inhibitors under clinical trials or approved by the US Food and Drug Administration (FDA) are either reversible or irreversible inhibitors that bind to the ATP-binding site in the kinase domain of ErbB receptors (Table 1). In addition to TKIs, several MAbs that target either EGFR (e.g., cetuximab), ErbB2 (trastuzumab), or ErbB2 dimerization (pertuzumab), are becoming major therapeutics, in particular for the ErbB2-positive breast cancer subtype where combination of anti-ErbB2, e.g., trastuzumab with conventional chemotherapy, has drastically changed the patients' prognosis and outcome (Piccart-Gebhart et al., 2005; Romond et al., 2005). As well, combination of MAbs with TKIs, e.g., trastuzumab plus anti-EGFR/anti-ErbB2 such as lapatinib or pertuzumab, revealed of clinical benefit for recurrent cancers following trastuzumab treatment for instance (Geyer et al., 2006; Joensuu et al., 2006). More recently, a double-blind placebo-controlled international multicentre clinical trial (CLEOPATRA)designed to evaluate the efficacy and safety of pertuzumab+trastuzumab+docetaxel (pertuzumab arm) *versus* placebo+trastuzumab+docetaxel (control arm) showed a survival improvement in the pertuzumab arm and also demonstrated that ErbB2 marker is suited for patient selection for the pertuzumab-based regimen in ErbB2-positive metastatic breast cancer or locally recurrent unresectable tumor (Baselga et al., 2014; Fleeman et al., 2015).

**Table 1:** **Representative FDA approved and experimental anti-ErbB therapeutic agents**.

**Substance name (Trade name)**	**Target**	**Type**	**FDA Indication (First approval date)**	**Mode of action**
Trastuzumab (Herclon®, Herceptin®)	ErbB2	Humanized IgG1 MAb^#^	ErbB2-positive breast and gastric carcinoma (1998)	Binds to domain IV of the extracellular domain of the ErbB2 (Carter et al., 1992)
Nimotuzumab	EGFR	Mouse/human chimeric IgG1 MAb	Glioma (Orphan designation, 2004)	Interacts with residues in the EGFR extracellular domain III (R353, S356, F357, T358, and H359T) (Mateo et al., 1997)
Cetuximab (Erbitux®)	EGFR	Mouse/human chimeric IgG1 MAb	Colorectal carcinoma, NSLCL, HNSCC^†^ (2006)	Binds exclusively to domain III of EGFR (e.g., I467, S468, Q408, and H409) leading to a partial occupancy of the ligand binding region and sterical prevention of receptor dimerization (Aboud-Pirak et al., 1988)
Panitumumab (Vectibix®)	EGFR	Humanized IgG2 MAb	Colorectal carcinoma (2006)	Binds to amino acid residues in domain III of EGFR (W386, E388, R390, and T391) leading to sterical prevention of receptor dimerization (Yang et al., 2001)
Pertuzumab (Perjeta®)	ErbB2	Humanized IgG1 MAb	ErbB2-positive breast cancer (2008)	Binds to ErbB2 near the center of domain II leading to a steric blockade of the binding pocket necessary for receptor dimerization (Adams et al., 2006)
Gefitinib (Iressa®)	EGFR	Small molecule	NSCLC^*^ (2003)	ATP-competitive TKI^‡^(Barker et al., 2001)
Erlotinib (Tarceva®)	EGFR	Small molecule	NSCLC, pancreatic carcinoma (2004)	Reversible ATP-competitive TKI (Moyer et al., 1997)
Lapatinib (Tykerb/Tyverb®)	EGFR, ErbB2	Small molecule	ErbB2-positive breast cancer (2007)	ATP-competitive TKI (Rusnak et al., 2001)
Vandetanib (Caprelsa®)	EGFR, RET, VEGFR	Small molecule	Medullary thyroid cancer (2011)	ATP-competitive TKI (Carlomagno et al., 2002)
Afatinib (Gilotrif®)	EGFR, ErbB2, ErbB4	Small molecule	NSCLC (2013)	Irreversible ATP-competitive TKI (Li et al., 2008)
Dacomitinib	EGFR, ErbB2, ErbB3, ErbB4	Small molecule	NSCLC (Phase III)	Irreversible pan-ErbB TKI (Engelman et al., 2007)
Varlitinib	EGFR, ErbB2	Small molecule	Advanced solid malignancies (Phase II)	ATP-competitive TKI; reversibly binds to EGFR and ErbB-2 and prevents their phosphorylation and activation (Miknis, 2005)
Sapitinib	EGFR, ErbB2, ErbB3	Small molecule	Advanced solid malignancies (Phase II)	Reversible ATP-competitive TKI (Hickinson et al., 2010)
Pelitinib	EGFR ErbB2, ErbB4	Small molecule	Colorectal carcinoma, NSCLC (Phase II)	Irreversible EGFR TKI (Wissner et al., 2003)
Canertinib	EGFR, ErbB2	Small molecule	NSCLC (Phase II)	Competitive TKI (Smaill et al., 2000)
Rociletinib	EGFR, ErbB2	Small molecule	NSCLC (Phase II)	Competitive TKI (Walter et al., 2013)

* *NSCLC, non-small cell lung carcinoma*.

† *HNSCC, Head and neck squamous cell carcinoma*.

‡ *TKI, Tyrosine kinase inhibitor*.

# *MAb, Monoclonal antibody*.

Despite of these successes, there remain major obstacles in achieving sustained response or cure with anti-ErbB inhibitors. The first obstacle refers to *de novo* or intrinsic resistance seen in patients expressing the ErbB targets yet failing to respond to anti-ErbB. This form of resistance is estimated to occur in up to ~20 and ~70% of ErbB2-positive patients with early and metastatic breast cancer treated with trastuzumab monotherapy, respectively (Harris et al., 2007; Wolff et al., 2007). The second type of resistance is the acquired form attributed to drug selection and can be seen in over 50% of patients who initially respond to anti-ErbB therapeutics but later become refractory to these drugs (Harris et al., 2007; Wolff et al., 2007).

Studies in preclinical models revealed intrinsic and acquired resistance to anti-ErbB therapeutics to involve multifactorial mechanisms both tumor- and host-related (Rexer and Arteaga, 2012). Briefly, mechanisms of primary drug resistance include emergence of pre-existing tumor cell subpopulations with (i) specific mutations in ErbB genes affecting the drug-target interaction; (ii) alternate splicing of ErbB gene leading to truncated isoforms of the receptors not recognized by the inhibitor, e.g., trastuzumab resistance in breast cancer has been associated with the expression of a truncated p95-ErbB2 receptor isoform that lacks trastuzumab antibody binding site; (iii) decreased MAb-induced cell-mediated cytotoxicity in ErbB2-positive cells such as due to an alteration in the binding of immune cells to Fc region of the MAb; and (iv) failure of MAb such as trastuzumab to induce ErbB2 receptor shedding, internalization, and/or degradation by ubiquitination (Rexer and Arteaga, 2012).

In contrast to intrinsic resistance, a broader range of mechanisms induced by drug pressure can mediate acquired resistance. These include secondary mutations that affect drug-ErbB target interaction (the most common are mutations in the TK domain), activation of compensatory signaling pathways able to bypass signaling blockade by the ErbB inhibitors, inefficient cellular transport/uptake of the drug, enhanced drug inactivation such as by phase II enzymes, up-regulation of survival signals, and altered drug pharmacokinetics and pharmacodistribution in the host. Targeting some of these mechanisms has provided alternative approaches to overcome resistance to anti-ErbB, e.g., combination of MAb such as trastuzumab with lapatinib or pertuzumab, the use of ado-trastuzumabemtansine (T-DM1), or combinations of trastuzumab with heat shock protein-90 (HSP90) inhibitors, PI3K inhibitors, and immune checkpoint modulators in combination with trastuzumab (Amiri-Kordestani et al., 2014).

In contrast to cancer-associated somatic mutations, single nucleotide polymorphisms (SNPs) are widespread in ErbB genes. In general, SNPs represent the most common genetic variations that occur in over 1.5% of healthy human population. In contrast somatic mutations are acquired genetic changes present in only a subset of cells. While most SNPs are silent with no apparent impact on physiological functions, some SNPs may impact on individual's susceptibility to anti-ErbB target therapeutics. The following chapters review the biological impact and predictive value of SNPs in ErbB genes.

## Potential impact of single nucleotide polymorphisms in ErbB genes on receptor activity, drug-receptor interaction and drug efficacy

SNPs are genetic variations that occur at a single position in a DNA sequence with fairly high frequency in the general population. SNPs are widespread in genes encoding all the 4 members of ErbB receptor family. Polymorphic point mutations could lead to variations in the amino acid sequence, however, SNPs can also occur in noncoding regions of DNA. In addition, mounting evidence support that genetic polymorphisms can also occur in microRNA-encoding sequences and this can contribute to phenotypic differences seen in earlier studies examining SNPs in relation to cancer via distinct regulatory mechanisms (Jin and Lee, 2013). Clearly, the contribution of SNPs in miRNA to pharmacogenetic in general requires in-depth investigations.

Genetic association studies have supported specific SNPs in ErbB genes to impact on ErbB biological activity and hence may represent potential markers for understanding individual susceptibility to anti-ErbB therapeutics. Below we are discussing the status of SNPs challenges to exploit these for optimization of the efficacy of anti-ErbB targeted therapeutics.

### EGFR

Several polymorphic variations in the promoter region of *EGFR* have been reported to impact on gene expression and function (Figure 1, Supplemental Table 1). The CA-simple sequence repeat 1 (CA-SSR1) is a highly polymorphic *locus* (14–21 CA dinucleotide repeats) situated in the first intron of *EGFR*. A lower CA-CSSR1 repeat number was found to modulate *EGFR* transcription *in vivo* and *in vitro* and correlated with increased transcription and protein expression (Gebhardt et al., 1999). The allele size distribution of CA-SSR1 demonstrates ethnic differences with East Asian having longer allele than individuals of European descent or African-Americans (Liu et al., 2011). Conversely, low CA-SSR1 number was associated with increased breast cancer risk in young women (Brandt et al., 2004), while a high CA-SSR1 number was correlated with better prognosis in luminal A breast cancer (Leite et al., 2014). Studies analyzing Asian population showed that the low CA repeat polymorphism is associated with better drug response and prolonged survival (Ichihara et al., 2007; Han et al., 2014). A second key polymorphism within EGFR pathways (R521K, previously assigned as R497K, rs2227983) is a single nucleotide change (G–A) in the codon 497 of the *EGFR* (Figure 1) leading to an arginine-lysine substitution in the extracellular domain of the subdomain IV. This variant is thought to decrease the ligand binding affinity of the receptor, thus dampening downstream impact of activation of downstream target such a *CMYC* and *VEGF* (Wang et al., 2007; Hsieh et al., 2012). R521K polymorphism has been associated with good prognostic features in breast cancer especially in patients with luminal A subtypes, as well as non-small cell lung, colorectal, and head and neck cancers (Wang et al., 2007; Sasaki et al., 2009; Hsieh et al., 2012; Leite et al., 2014). Other studies have reported that R521K polymorphism is associated with favorable outcome in cetuximab-based and 5-FU-based chemotherapy but shows negative correlation in gefitinib-based chemotherapy (Wang et al., 2007; Lurje et al., 2008; Sasaki et al., 2009; Dahan et al., 2011; Hsieh et al., 2012; Leite et al., 2014). This indicate that SNP in individual gene, whether it would affect cancer cell proliferation or not, may influence the chromosome instability (CIN) and even that cancer progression is not altered remarkably it can result different profiles of clinical response to chemotherapy; however, further investigation still is necessary and the field is very promising.

Recent genome-wide studies (GWAS) have identified an EGFR polymorphisms (rs2252586 and rs11979158) to be associated with increased glioma risk (Sanson et al., 2011). Two further polymorphisms in the promoter region of EGFR, SNPs -216 (rs712829) and -191 (rs712830), were associated with increased protein synthesis; these SNPs are rare in East Asian populations compared to other ethnicities (Liu et al., 2011). SNPs -216 variant is located -216 upstream from the initiator ATG and the change of nucleoside is guanine to thymine (G/T or T/T) in a important binding site for the transcription factor SP1 that is necessary for activation of *EGFR* promoter activity (Liu et al., 2005). This variant is frequent in individuals of European descent and African-American than Asians (Liu et al., 2005) and has been associated with increased intrinsic gene expression (Liu et al., 2011). Furthermore, patients harboring SNP-216 and CA-SSR1 germline polymorphisms were more likely to present somatic mutations of *EGFR*, in particular microdeletions of exon 19. These deletions are common in lung cancer and were reported to strongly predict response to TKI (Liu et al., 2011).

**Figure 2:**
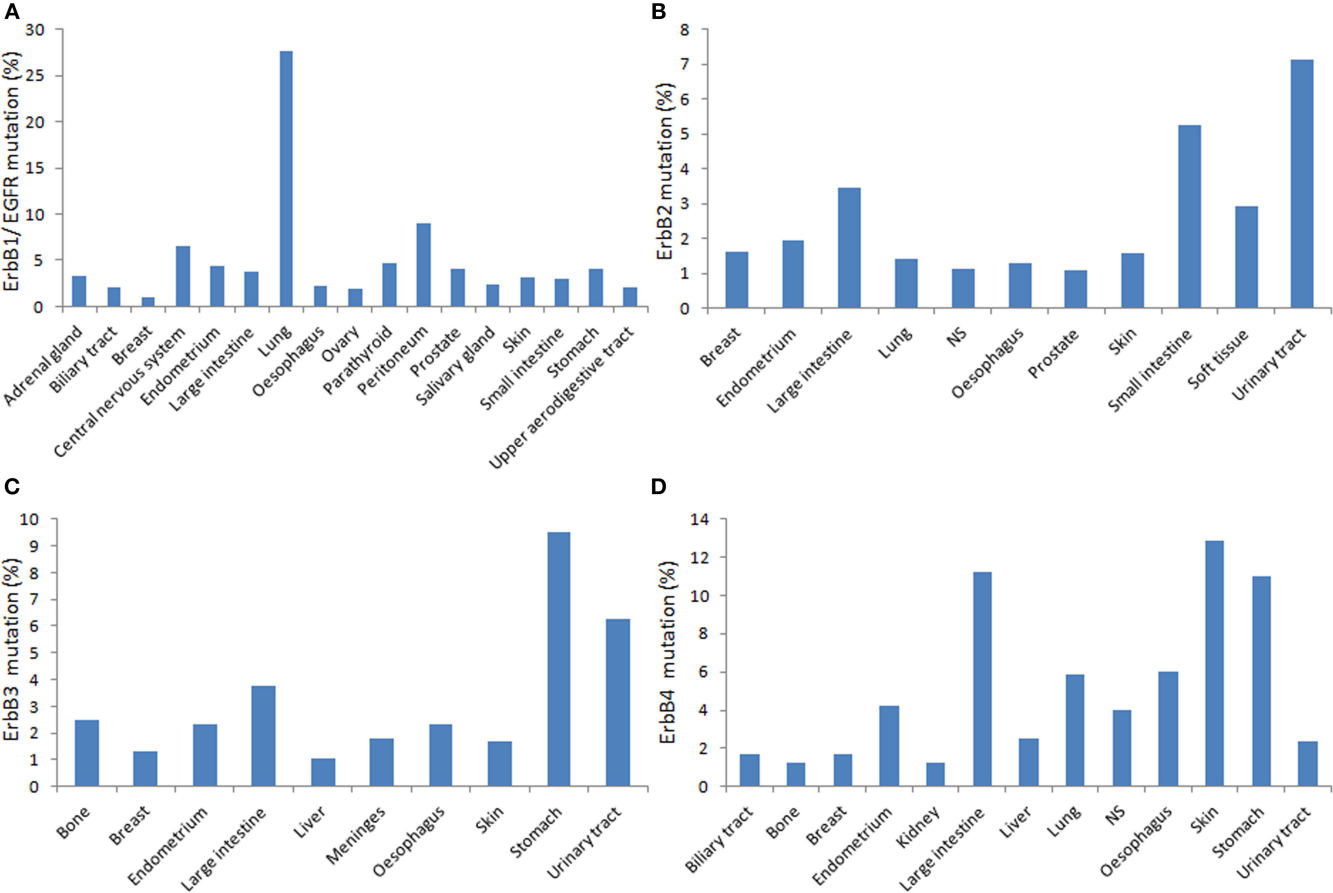
**Distribution of the relative percentage of somatic mutations among different cancers types according to the cataloge of somatic mutations in cancer (COSMIC) database in human primary tumors**. Each panel shows mutations rates for corresponding ErbB (**A**. EGFR; **B**. ErbB2; **C**. ErbB3; **D**. ErbbB4) per tumor tissue affected (http://cancer.sanger.ac.uk/cancergenome/projects/cosmic/).

Besides polymorphic variations, somatic mutations identified in ErbB genes, including EGFR (Figures 1, 2) greatly influence in the receptor activity and interaction with TKIs, it is of fundamental importance to investigate the cooperation between SNPs and cancer-associated somatic mutations in ErbB genes to better understand the utility of ErbB polymorphisms to predict individual susceptibility to anti-ErbB therapeutics.

### ERBB2

The most investigated *ERBB2* polymorphism related to cancer risk is I655V (rs1136201) located at the codon 655 (ATC/isoleucine to GTC/valine) in the transmembrane domain of the receptor (Figure 1, Supplemental Table 2) (Kuraoka et al., 2003; Puputti et al., 2006; Han et al., 2014). The amino acid change could result in increased protein tyrosine kinase activity. Tumors harboring the I655V polymorphism showed higher tumorigenic potential in preclinical models and breast cancers expressing this variant also manifest an aggressive phenotype (Han et al., 2014). I655Vpolymorphism confers a higher risk of trastuzumab-associated cardiotoxicity but does not directly affect patients' survival, and hence it may be useful for the prevention of cardiovascular events in patient receiving after therapy (Beauclair et al., 2007). Further, a systematic review and meta-analysis studying ErbB2 polymorphism in over 45,000 breast cancer patients showed that there is a lack of experiments replication for association studies between the polymorphic variants and cancer risk, as well as lack of quality control of genotyping assays or publication bias influencing the results (Dahabreh and Murray, 2011).

### ERBB3

Compared to the other members of the ErbB family, ErbB3 receptor has been under-investigated in part because it was initially identified as an inactive kinase receptor whose function depends on interaction with ErbB partners, primarily through heterodimerization. Therefore, functional polymorphisms in *ERBB3* were long neglected for this pseudo-kinase (Shi et al., 2010). Recent studies revealed at least 1091 polymorphic forms in human *ERBB3* gene (dbSNP database; http://www.ncbi.nlm.nih.gov/snp) (Figure 1, Supplemental Table 3). However, only one study reported clinical relevance of the polymorphism 276C/T (rs2271188) in the promoter region of the *ERBB3*. This variant affected gene expression contributing to genetic susceptibility and was associated with higher risk of lung cancer in never-smokers (Sung et al., 2012). Noticeable, one research group reported the association of ErbB3 overexpression with resistance to tamoxifen as well as to small molecule TKI in breast cancer; however, a possible contribution of SNPs was not addressed (Liu et al., 2007).

### ERBB4

Multiple SNPs in the *ERBB4* promoter region were associated with adverse clinical features (Figure 1, Supplemental Table 4). The frequency and prognostic significance of two *ERBB4* promoter region variants, -782G/T (rs62626348) and -815A/T (rs62626347), were investigated in a large cohort of breast cancer patients. 782G/T conferred highest cancer risk (Rokavec et al., 2007) but another study observed an association of this variant with well-differentiated breast tumor (Kurppa et al., 2014). The variant815A/T was correlated with poor survival and proposed as a prognostic marker in high-risk early breast cancer (Kurppa et al., 2014). A genome-wide association study (GWAS) identified a breast cancer risk variant in *ERBB4* at 2q34 (rs13393577) to occur in European and Chinese populations. However, the impact of ErbB4 polymorphisms on response to anti-ErbB therapeutics remains to be established.

## Conclusion and perspectives

Anti-ErbB drugs are becoming cornerstone therapeutics for many cancers and in particular for lung and breast cancers subtype where combination of anti-ErbB with conventional chemotherapy has drastically changed the patient's outcome. Yet a dilemma for the oncologist is how to predict with certainty patients likely will respond to primary treatment from those that may be intrinsically resistant despite the presence of the ErbB target. With the advent of the genome-wide scanning technologies, remarkable advances have been made in the identification of single polymorphic variants in ErbB genes. However, the significance of most SNPs leading to amino acid substitutions to ErbB receptor signaling and cell susceptibility to anti-ErbB therapeutics remains to be established. With the availability of technologies to manipulate gene expression in intact cells, such as the clustered regularly interspaced short palindromic repeat (CRISPER) system, allow to develop cell models expressing wild-type *versus* polymorphic variants for functional studies, which are essential for surveying the implications of ErbB SNPs landscape in greater details, in particular in relation to receptor activity, receptor topology, and response to anti-ErbB therapeutics. This represents the backbone to draw testable hypotheses on SNP-impacts on receptor structure activity and interaction with the inhibitor. This knowledge can be later exploited for the design of more meaningful large-scale SNP association studies. In this respect, among challenging questions is that relevant SNPs may likely produce borderline resistance or sensitivity to anti-ErbB, which is difficult to investigate in the clinical setting. Alternatives for standard clinical trials such as clinical trial simulations may be required to fill this gap and ultimately design alternative molecules that target relevant SNP genetic variants. In summary, identification of ErbB genetic variations that can predict drug response or resistance is a first step toward exploiting the utility of pharmacogenetics to tailor individual ErbB-based therapy regimens, a prerequisite to optimize care cancer.

## Author contributions

Original idea and study design by Moulay A. Alaoui-Jamali. Figures by Sabrina Daniela da Silva, Moulay A. Alaoui-Jamali, Grégoire B. Morand, and Sabrina Daniela da Silva all have actively participated to manuscript drafting, elaboration of tables and approved the final version.

### Conflict of interest statement

The authors declare that the research was conducted in the absence of any commercial or financial relationships that could be construed as a potential conflict of interest.
