# Supplementary material for: ErbB polymorphisms: insights and implications for response to targeted cancer therapeutics
Source: Front Genet. 2015 Feb 4;6:17. doi: 10.3389/fgene.2015.00017 (PMC4316710; doi:10.3389/fgene.2015.00017)
Supplement: Supplementary file 1 [file Table1.PDF]

11. Supplemental material

Supplemental Table 1: EGFR Protein changing polymorphism according to NCBI SNP Viewer

VariantID	Genomic position (chr. 7)	Transcript change	Protein change
rs373129709	55019338	c.61G>T	Ala21Ser
rs144158123	55142298	c.101C>G	Thr34Arg
rs375919121	55142313	c.116C>T	Thr39Met
rs147740818	55142324	c.127A>G	Thr43Ala
rs369580836	55142409	c.212A>G	Gln71Arg
rs374986786	55143305	c.241A>G	Thr81Ala
rs142061256	55143333	c.269T>A	Leu90His
rs35515689	55143347	c.283A>C	Thr95Pro
rs17289589	55143357	c.293G>A	Arg98Gln
rs376963968	55143363	c.299C>A	Pro100His
rs145113601	55143390	c.326G>C	Gly109Ala
rs377444977	55143443	c.379G>A	Ala127Thr
rs370744986	55152639	c.722C>T	Thr241Ile
rs200664836	55152648	c.731G>A	Arg244Gln
rs374084791	55154021	c.758A>G	Lys253Arg
rs372202099	55154027	c.764G>A	Arg255Gln
rs138847501	55154032	c.769G>A	Glu257Lys
rs17336639	55154060	c.797C>G	Pro266Arg
rs17336639	55154060	c.797C>A	Pro266Gln
rs199796955	55154107	c.844G>A	Glu282Lys
rs149840192	55154129	c.866C>T	Ala289Val
rs150549265	55154137	c.874G>A	Val292Met
rs149321481	55155878	c.938C>T	Ala313Val
rs367680488	55155908	c.968T>C	Val323Ala
rs144460286	55155922	c.982A>C	Lys328Gln
rs139429793	55155928	c.988G>A	Glu330Lys
rs371234907	55156569	c.1043C>T	Ser348Leu
rs201364864	55157731	c.1276A>G	Ile426Val
rs372990493	55160173	c.1333A>G	Ile445Val
rs377567759	55160191	c.1351C>T	Arg451Cys
rs146711874	55160214	c.1374T>G	Asp458Glu
rs200592648	55160222	c.1382T>C	Val461Ala
rs371114444	55161532	c.1532C>A	Ser511Tyr
rs368484180	55161552	c.1552C>T	Pro518Ser
rs116057045	55161557	c.1557G>T	Glu519Asp
rs2227983	55161562	c.1562G>A	Arg521Lys
rs2227983	55161562	c.1562G>T	Arg521Met
rs2227983	55161562	c.1562G>C	Arg521Thr
rs150477666	55161580	c.1580G>A	Arg527Gln
rs370810719	55163822	c.1721G>A	Arg574Gln
rs144943614	55165331	c.1774G>A	Val592Ile
rs28384375	55165332	c.1775T>C	Val592Ala
rs371483915	55165334	c.1777A>C	Lys593Gln
rs139236063	55165350	c.1793G>T	Gly598Val
rs149375515	55165356	c.1799T>C	Met600Thr
rs201498575	55165359	c.1802G>A	Gly601Glu
rs143152775	55165373	c.1816C>G	Leu606Val
rs201061916	55165388	c.1831G>A	Ala611Thr

rs150899403	55165416	c.1859G>A	Cys620Tyr
rs28384376	55165428	c.1871G>T	Cys624Phe
rs371814116	55170312	c.1886G>A	Gly629Glu
rs369826866	55170323	c.1897C>A	Leu633Ile
rs147139524	55170329	c.1903G>T	Ala635Ser
rs140443314	55170344	c.1918C>A	Leu640Ile
rs371909721	55170381	c.1955G>T	Gly652Val
rs145830434	55170401	c.1975G>A	Gly659Arg
rs374459674	55170436	c.2010G>T	Trp670Cys
rs138963326	55170449	c.2023C>G	Leu675Val
rs201419843	55170466	c.2040G>C	Gln680His
rs111275056	55170483	c.2057G>A	Cys686Tyr
rs368763603	55170525	c.2099C>T	Thr700Met
rs77402685	55170533	c.2107T>C	Ser703Pro
rs10258568	55170534	c.2108C>T	Ser703Phe
rs200989322	55171212	c.1918G>T	Gly640Trp
rs140516819	55172999	c.1936A>C	Ile646Leu
rs201580890	55173038	c.1975G>C	Val659Leu
rs76946721	55173066	c.2003T>G	Met668Arg
rs17337079	55173083	c.2020G>A	Val674Ile
rs150423237	55173087	c.2024G>A	Arg675Gln
rs138193597	55173096	c.2033C>T	Thr678Met
rs369399038	55173101	c.2038C>T	Arg680Trp
rs373336251	55173102	c.2039G>A	Arg680Gln
rs397517083	55173924	c.2065G>C	Val689Leu
rs397517084	55173976	c.2117T>C	Ile706Thr
rs397517085	55173985	c.2126A>C	Glu709Ala
rs373578289	55173997	c.2138A>C	Lys713Thr
rs28929495	55174014	c.2155G>C	Gly719Arg
rs28929495	55174014	c.2155G>T	Gly719Cys
rs28929495	55174014	c.2155G>A	Gly719Ser
rs121913428	55174015	c.2156G>C	Gly719Ala
rs121913428	55174015	c.2156G>A	Gly719Asp
rs138240620	55174039	c.2180A>G	Tyr727Cys
rs121913434	55174725	c.2188C>T	Leu730Phe
rs121913467	55174730	c.2193G>A	Trp731Ter
rs121913446	55174735	c.2198C>T	Pro733Leu
rs121913420	55174737	c.2200G>A	Glu734Lys
rs121913430	55174740	c.2203G>A	Gly735Ser
rs397517092	55174756	c.2219T>C	Ile740Thr
rs121913466	55174762	c.2225T>C	Val742Ala
rs121913433	55174771	c.2234A>G	Lys745Arg
rs121913427	55174773	c.2236G>A	Glu746Lys
rs397517097	55174777	c.2240T>C	Leu747Ser
rs121913229	55174785	c.2248G>C	Ala750Pro
rs121913464	55174792	c.2255C>A	Ser752Tyr
rs121913231	55174794	c.2257C>T	Pro753Ser
rs397517101	55174797	c.2260A>G	Lys754Glu
rs397517102	55174807	c.2270A>T	Lys757Met
rs121913418	55174818	c.2281G>A	Asp761Asn
rs121913418	55174818	c.2281G>T	Asp761Tyr
rs374873413	55181302	c.2293G>C	Val765Leu
rs397517107	55181309	c.2300C>T	Ala767Val
rs121913465	55181312	c.2303G>T	Ser768Ile

rs147149347	55181314	c.2305G>T	Val769Leu
rs121913432	55181327	c.2318A>G	His773Arg
rs397517119	55181344	c.2335G>T	Gly779Cys
rs397517120	55181345	c.2336G>T	Gly779Val
rs121434569	55181378	c.2369C>T	Thr790Met
rs370289230	55181389	c.2380C>A	Pro794Thr
rs121913230	55181437	c.2428G>A	Gly810Ser
rs121913431	55181438	c.2429G>A	Gly810Asp
rs397517125	55181473	c.2464G>A	Ala822Thr
rs150749913	55191729	c.2480A>T	Tyr827Phe
rs371228501	55191740	c.2491C>T	Arg831Cys
rs150036236	55191741	c.2492G>A	Arg831His
rs397517126	55191746	c.2497T>G	Leu833Val
rs397517127	55191749	c.2500G>T	Val834Leu
rs397517128	55191753	c.2504A>T	His835Leu
rs374952732	55191755	c.2506C>T	Arg836Cys
rs146121458	55191756	c.2507G>A	Arg836His
rs143884981	55191767	c.2518G>A	Ala840Thr
rs146795390	55191776	c.2527G>A	Val843Ile
rs148934350	55191792	c.2543C>T	Pro848Leu
rs121913443	55191821	c.2572C>A	Leu858Met
rs121434568	55191822	c.2573T>G	Leu858Arg
rs397517130	55191829	c.2580A>T	Lys860Asn
rs121913444	55191831	c.2582T>G	Leu861Arg
rs121913444	55191831	c.2582T>A	Leu861Gln
rs397517132	55191846	c.2597A>T	Glu866Val
rs104886013	55191851	c.2602G>A	Glu868Lys
rs397517134	55191861	c.2612C>G	Ala871Gly
rs397517136	55192782	c.2642T>C	Met881Thr
rs397517137	55192794	c.2654C>T	Ser885Leu
rs151064287	55192815	c.2675C>T	Thr892Ile
rs376176117	55192839	c.2699A>T	Tyr900Phe
rs376822837	55198761	c.2746G>A	Asp916Asn
rs368698152	55198854	c.2839A>G	Met947Val
rs201830126	55200330	c.2863G>A	Ala955Thr
rs104886026	55200333	c.2866G>A	Asp956Asn
rs17337451	55200351	c.2884C>G	Arg962Gly
rs144496976	55200352	c.2885G>A	Arg962His
rs1140476	55200396	c.2929C>T	Arg977Cys
rs17290699	55201204	c.2963A>C	His988Pro
rs149248025	55201237	c.2996G>A	Arg999His
rs148019583	55201266	c.3025G>A	Asp1009Asn
rs182857647	55201327	c.3086C>T	Thr1029Met
rs34352568	55201342	c.3101T>G	Leu1034Arg
rs375035197	55201748	c.3128A>G	Asn1043Ser
rs142442994	55201759	c.3139G>A	Val1047Met
rs78244461	55201763	c.3143C>T	Ala1048Val
rs374501041	55202557	c.3203G>A	Arg1068Gln
rs184614596	55202591	c.3237G>C	Glu1079Asp
rs371229748	55202598	c.3244A>T	Ile1082Leu
rs373990043	55202603	c.3249C>A	Asp1083Glu
rs367870311	55202622	c.3268C>T	Pro1090Ser
rs139388758	55205291	c.3307G>A	Gly1103Ser
rs376598259	55205294	c.3310T>C	Ser1104Pro

rs369498625	55205318	c.3334A>C	Asn1112His
rs199738264	55205367	c.3383C>T	Pro1128Leu
rs145189325	55205411	c.3427C>G	Gln1143Glu
rs368892932	55205438	c.3454G>C	Asp1152His
rs149174093	55205451	c.3467A>C	His1156Pro
rs140028234	55205456	c.3472G>C	Ala1158Pro
rs41321844	55205469	c.3485G>A	Ser1162Asn
rs147896627	55205502	c.3518A>C	Gln1173Pro
rs199661469	55205531	c.3547C>T	Pro1183Ser
rs372948989	55205556	c.3572C>T	Thr1191Ile
rs369585356	55205585	c.3601G>A	Ala1201Thr
rs201717672	55205586	c.3602C>T	Ala1201Val
rs35918369	55205613	c.3629C>T	Ala1210Val

Supplemental Table 2: ErbB2 Protein changing polymorphism according to NCBI SNP Viewer

VariantID	Genomic position (Chr 17)	Transcript change	Protein change
rs149937802	39707016	c.10C>T	Arg4Trp
rs4252596	39699581	c.22C>A	Pro8Thr
rs193171026	39700281	c.43C>T	Leu15Phe
rs144019910	39707056	c.50G>A	Arg17His
rs149937802	39707016	c.55C>T	Arg19Trp
rs140441229	39707086	c.80A>G	Gln27Arg
rs144019910	39707056	c.95G>A	Arg32His
rs149937802	39707016	c.100C>T	Arg34Trp
rs140441229	39707086	c.125A>G	Gln42Arg
rs144019910	39707056	c.140G>A	Arg47His
rs61737968	39708331	c.146A>C	Glu49Ala
rs376524324	39708345	c.160G>A	Val54Met
rs140441229	39707086	c.170A>G	Gln57Arg
rs61737968	39708331	c.191A>C	Glu64Ala
rs376524324	39708345	c.205G>A	Val69Met
rs61737968	39708331	c.236A>C	Glu79Ala
rs376524324	39708345	c.250G>A	Val84Met
rs141135746	39708459	c.274C>G	Pro92Ala
rs370959592	39708460	c.275C>T	Pro92Leu
rs373824622	39708478	c.293C>G	Pro98Arg
rs200208742	39708505	c.320T>C	Leu107Pro
rs141135746	39708459	c.319C>G	Pro107Ala
rs370959592	39708460	c.320C>T	Pro107Leu
rs193171026	39700281	c.334C>T	Leu112Phe

VariantID	Genomic position (Chr 17)	Transcript change	Protein change
rs185670819	39708523	c.338G>A	Arg113Gln
rs373824622	39708478	c.338C>G	Pro113Arg
rs200963868	39709330	c.362G>T	Gly121Val
rs151122410	39709333	c.365G>C	Gly122Ala
rs200208742	39708505	c.365T>C	Leu122Pro
rs141135746	39708459	c.364C>G	Pro122Ala
rs370959592	39708460	c.365C>T	Pro122Leu
rs185670819	39708523	c.383G>A	Arg128Gln
rs373824622	39708478	c.383C>G	Pro128Arg
rs149937802	39707016	c.391C>T	Arg131Trp
rs200963868	39709330	c.407G>T	Gly136Val
rs151122410	39709333	c.410G>C	Gly137Ala
rs200208742	39708505	c.410T>C	Leu137Pro
rs185670819	39708523	c.428G>A	Arg143Gln
rs144019910	39707056	c.431G>A	Arg144His
rs200963868	39709330	c.452G>T	Gly151Val
rs151122410	39709333	c.455G>C	Gly152Ala
rs140441229	39707086	c.461A>G	Gln154Arg
rs113619125	39709828	c.500C>T	Pro167Leu
rs61737968	39708331	c.527A>C	Glu176Ala
rs376524324	39708345	c.541G>A	Val181Met
rs113619125	39709828	c.545C>T	Pro182Leu
rs113619125	39709828	c.590C>T	Pro197Leu
rs376183465	39710176	c.644C>T	Thr215Met

rs141135746	39708459	c.655C>G	Pro219Ala
rs370959592	39708460	c.656C>T	Pro219Leu
rs373824622	39708478	c.674C>G	Pro225Arg
rs376183465	39710176	c.689C>T	Thr230Met
rs200208742	39708505	c.701T>C	Leu234Pro
rs200382130	39710388	c.718G>T	Ala240Ser
rs185670819	39708523	c.719G>A	Arg240Gln
rs376183465	39710176	c.734C>T	Thr245Met
rs200963868	39709330	c.743G>T	Gly248Val
rs151122410	39709333	c.746G>C	Gly249Ala
rs200382130	39710388	c.763G>T	Ala255Ser
rs149210045	39710436	c.766G>A	Glu256Lys
rs200382130	39710388	c.808G>T	Ala270Ser
rs149210045	39710436	c.811G>A	Glu271Lys
rs149210045	39710436	c.856G>A	Glu286Lys
rs113619125	39709828	c.881C>T	Pro294Leu
rs373474372	39712379	c.989A>T	Asn330Ile
rs376183465	39710176	c.1025C>T	Thr342Met
rs373474372	39712379	c.1034A>T	Asn345Ile
rs148068883	39712438	c.1048A>G	Ser350Gly
rs141116145	39715294	c.1067C>A	Ala356Asp
rs373474372	39712379	c.1079A>T	Asn360Ile
rs148068883	39712438	c.1093A>G	Ser365Gly
rs200382130	39710388	c.1099G>T	Ala367Ser
rs141116145	39715294	c.1112C>A	Ala371Asp
rs377649991	39715344	c.1117C>A	Leu373Met
rs148068883	39712438	c.1138A>G	Ser380Gly
rs149210045	39710436	c.1147G>A	Glu383Lys
rs141116145	39715294	c.1157C>A	Ala386Asp
rs377649991	39715344	c.1162C>A	Leu388Met
rs199668084	39715490	c.1177A>G	Ser393Gly
rs142783371	39715493	c.1180G>A	Val394Ile
rs367606199	39715517	c.1204C>T	Arg402Trp
rs377649991	39715344	c.1207C>A	Leu403Met
rs147382623	39715524	c.1211G>A	Arg404Gln
rs199668084	39715490	c.1222A>G	Ser408Gly
rs142783371	39715493	c.1225G>A	Val409Ile
rs367606199	39715517	c.1249C>T	Arg417Trp
rs147382623	39715524	c.1256G>A	Arg419Gln
rs4252633	39715782	c.1266G>T	Trp422Cys
rs199668084	39715490	c.1267A>G	Ser423Gly
rs142783371	39715493	c.1270G>A	Val424Ile
rs200497646	39715792	c.1276C>T	Arg426Cys
rs367606199	39715517	c.1294C>T	Arg432Trp
rs373192991	39715811	c.1295G>A	Gly432Asp
rs147382623	39715524	c.1301G>A	Arg434Gln
rs4252633	39715782	c.1311G>T	Trp437Cys
rs201097345	39715835	c.1319A>G	His440Arg
rs200497646	39715792	c.1321C>T	Arg441Cys
rs373192991	39715811	c.1340G>A	Gly447Asp
rs201021373	39715862	c.1346C>T	Thr449Met
rs370565888	39715868	c.1352C>T	Pro451Leu
rs4252633	39715782	c.1356G>T	Trp452Cys
rs201097345	39715835	c.1364A>G	His455Arg

rs200497646	39715792	c.1366C>T	Arg456Cys
rs371450390	39715886	c.1370G>A	Arg457Gln
rs375382055	39715885	c.1369C>T	Arg457Trp
rs373474372	39712379	c.1370A>T	Asn457Ile
rs142456637	39715892	c.1376C>T	Pro459Leu
rs373192991	39715811	c.1385G>A	Gly462Asp
rs201021373	39715862	c.1391C>T	Thr464Met
rs370565888	39715868	c.1397C>T	Pro466Leu
rs145762641	39715915	c.1399G>A	Ala467Thr
rs182572604	39715922	c.1406G>A	Arg469Gln
rs199530208	39715921	c.1405C>T	Arg469Trp
rs201097345	39715835	c.1409A>G	His470Arg
rs371450390	39715886	c.1415G>A	Arg472Gln
rs375382055	39715885	c.1414C>T	Arg472Trp
rs142456637	39715892	c.1421C>T	Pro474Leu
rs148068883	39712438	c.1429A>G	Ser477Gly
rs201021373	39715862	c.1436C>T	Thr479Met
rs370565888	39715868	c.1442C>T	Pro481Leu
rs145762641	39715915	c.1444G>A	Ala482Thr
rs141116145	39715294	c.1448C>A	Ala483Asp
rs182572604	39715922	c.1451G>A	Arg484Gln
rs199530208	39715921	c.1450C>T	Arg484Trp
rs371623072	39716333	c.1456G>A	Ala486Thr
rs371450390	39715886	c.1460G>A	Arg487Gln
rs375382055	39715885	c.1459C>T	Arg487Trp
rs142456637	39715892	c.1466C>T	Pro489Leu
rs202202058	39716354	c.1477C>T	Pro493Ser
rs145762641	39715915	c.1489G>A	Ala497Thr
rs182572604	39715922	c.1496G>A	Arg499Gln
rs199530208	39715921	c.1495C>T	Arg499Trp
rs377649991	39715344	c.1498C>A	Leu500Met
rs371623072	39716333	c.1501G>A	Ala501Thr
rs140711914	39716378	c.1501T>C	Cys501Arg
rs140980495	39716394	c.1517G>A	Arg506Gln
rs202202058	39716354	c.1522C>T	Pro508Ser
rs150203173	39716421	c.1544G>A	Arg515Gln
rs371623072	39716333	c.1546G>A	Ala516Thr
rs140711914	39716378	c.1546T>C	Cys516Arg
rs199668084	39715490	c.1558A>G	Ser520Gly
rs140980495	39716394	c.1562G>A	Arg521Gln
rs142783371	39715493	c.1561G>A	Val521Ile
rs202202058	39716354	c.1567C>T	Pro523Ser
rs367606199	39715517	c.1585C>T	Arg529Trp
rs150203173	39716421	c.1589G>A	Arg530Gln
rs147382623	39715524	c.1592G>A	Arg531Gln
rs140711914	39716378	c.1591T>C	Cys531Arg
rs201470725	39716553	c.1595C>T	Pro532Leu
rs140980495	39716394	c.1607G>A	Arg536Gln
rs150203173	39716421	c.1634G>A	Arg545Gln
rs201470725	39716553	c.1640C>T	Pro547Leu
rs4252633	39715782	c.1647G>T	Trp549Cys
rs200497646	39715792	c.1657C>T	Arg553Cys
rs373192991	39715811	c.1676G>A	Gly559Asp
rs201470725	39716553	c.1685C>T	Pro562Leu

rs201097345	39715835	c.1700A>G	His567Arg
rs145409713	39717375	c.1703C>A	Ala568Asp
rs145409713	39717375	c.1703C>G	Ala568Gly
rs369903296	39717377	c.1705C>T	Arg569Cys
rs201021373	39715862	c.1727C>T	Thr576Met
rs370565888	39715868	c.1733C>T	Pro578Leu
rs377370642	39717413	c.1741A>G	Met581Val
rs145409713	39717375	c.1748C>A	Ala583Asp
rs145409713	39717375	c.1748C>G	Ala583Gly
rs369903296	39717377	c.1750C>T	Arg584Cys
rs371450390	39715886	c.1751G>A	Arg584Gln
rs375382055	39715885	c.1750C>T	Arg584Trp
rs142456637	39715892	c.1757C>T	Pro586Leu
rs145762641	39715915	c.1780G>A	Ala594Thr
rs182572604	39715922	c.1787G>A	Arg596Gln
rs199530208	39715921	c.1786C>T	Arg596Trp
rs377370642	39717413	c.1786A>G	Met596Val
rs145409713	39717375	c.1793C>A	Ala598Asp
rs145409713	39717375	c.1793C>G	Ala598Gly
rs369903296	39717377	c.1795C>T	Arg599Cys
rs199726056	39717480	c.1808C>T	Ser603Leu
rs199726056	39717480	c.1808C>T	Ser603Phe
rs377370642	39717413	c.1831A>G	Met611Val
rs371623072	39716333	c.1837G>A	Ala613Thr
rs367599823	39719816	c.1838C>G	Pro613Arg
rs370514427	39719821	c.1843G>A	Glu615Lys
rs201784472	39719828	c.1850G>C	Arg617Thr
rs199726056	39717480	c.1853C>T	Ser618Phe
rs202202058	39716354	c.1858C>T	Pro620Ser
rs1801201	39723332	c.1870A>G	Ile624Val
rs1136201	39723335	c.1873A>G	Ile625Val
rs140711914	39716378	c.1882T>C	Cys628Arg
rs367599823	39719816	c.1883C>G	Pro628Arg
rs370514427	39719821	c.1888G>A	Glu630Lys
rs201784472	39719828	c.1895G>C	Arg632Thr
rs140980495	39716394	c.1898G>A	Arg633Gln
rs199726056	39717480	c.1898C>T	Ser633Phe
rs372616729	39723374	c.1912G>C	Gly638Arg
rs1801201	39723332	c.1915A>G	Ile639Val
rs1136201	39723335	c.1918A>G	Ile640Val
rs150203173	39716421	c.1925G>A	Arg642Gln
rs367599823	39719816	c.1928C>G	Pro643Arg
rs370514427	39719821	c.1933G>A	Glu645Lys
rs201784472	39719828	c.1940G>C	Arg647Thr
rs372616729	39723374	c.1957G>C	Gly653Arg
rs1801201	39723332	c.1960A>G	Ile654Val
rs1136201	39723335	c.1963A>G	Ile655Val
rs201470725	39716553	c.1976C>T	Pro659Leu
rs372616729	39723374	c.2002G>C	Gly668Arg
rs34602395	39723560	c.2018G>T	Ser673Ile
rs34602395	39723560	c.2063G>T	Ser688Ile
rs145409713	39717375	c.2084C>A	Ala695Asp
rs145409713	39717375	c.2084C>G	Ala695Gly
rs369903296	39717377	c.2086C>T	Arg696Cys

rs377013455	39717146	c.2108G>A	Arg703Gln
rs34602395	39723560	c.2108G>T	Ser703Ile
rs143047248	39717152	c.2114G>T	Arg705Met
rs377370642	39717413	c.2122A>G	Met708Val
rs121913470	39723967	c.2174T>C	Leu725Ser
rs199726056	39717480	c.2189C>T	Ser730Leu
rs199726056	39717480	c.2189C>T	Ser730Phe
rs1801201	39723332	c.2203A>G	Ile735Val
rs1136201	39723335	c.2206A>G	Ile736Val
rs56366519	39724006	c.2213T>C	Leu738Ser
rs121913468	39724008	c.2215G>C	Asp739His
rs121913470	39723967	c.2219T>C	Leu740Ser
rs372616729	39723374	c.2245G>C	Gly749Arg
rs56366519	39724006	c.2258T>C	Leu753Ser
rs121913468	39724008	c.2260G>C	Asp754His
rs121913470	39723967	c.2264T>C	Leu755Ser
rs56366519	39724006	c.2303T>C	Leu768Ser
rs121913468	39724008	c.2305G>C	Asp769His
rs34602395	39723560	c.2351G>T	Ser784Ile
rs121913470	39723967	c.2507T>C	Leu836Ser
rs56366519	39724006	c.2546T>C	Leu849Ser
rs121913468	39724008	c.2548G>C	Asp850His
rs201374810	39727308	c.3083A>C	Asp1028Ala
rs141142822	39727317	c.3092T>C	Leu1031Pro
rs376450229	39727325	c.3100G>A	Glu1034Lys
rs201374810	39727308	c.3128A>C	Asp1043Ala
rs202149348	39727353	c.3128C>G	Ser1043Cys
rs141142822	39727317	c.3137T>C	Leu1046Pro
rs376450229	39727325	c.3145G>A	Glu1049Lys
rs200796676	39727385	c.3160G>T	Asp1054Tyr
rs201374810	39727308	c.3173A>C	Asp1058Ala
rs202149348	39727353	c.3173C>G	Ser1058Cys
rs141142822	39727317	c.3182T>C	Leu1061Pro
rs376450229	39727325	c.3190G>A	Glu1064Lys
rs373724781	39727423	c.3198G>C	Lys1066Asn
rs200796676	39727385	c.3205G>T	Asp1069Tyr
rs200455249	39727440	c.3215C>T	Pro1072Leu
rs202149348	39727353	c.3218C>G	Ser1073Cys
rs111611886	39727448	c.3223G>A	Asp1075Asn
rs143958183	39727466	c.3241C>T	Arg1081Trp
rs373724781	39727423	c.3243G>C	Lys1081Asn
rs200796676	39727385	c.3250G>T	Asp1084Tyr
rs200455249	39727440	c.3260C>T	Pro1087Leu
rs111611886	39727448	c.3268G>A	Asp1090Asn
rs148211805	39727510	c.3285T>A	Asp1095Glu
rs143958183	39727466	c.3286C>T	Arg1096Trp
rs373724781	39727423	c.3288G>C	Lys1096Asn
rs200455249	39727440	c.3305C>T	Pro1102Leu
rs111611886	39727448	c.3313G>A	Asp1105Asn
rs150165942	39727538	c.3313C>T	Pro1105Ser
rs148211805	39727510	c.3330T>A	Asp1110Glu
rs143958183	39727466	c.3331C>T	Arg1111Trp
rs146177313	39727699	c.3333C>A	Asn1111Lys
rs138611862	39727706	c.3340G>C	Asp1114His

rs150165942	39727538	c.3358C>T	Pro1120Ser
rs148211805	39727510	c.3375T>A	Asp1125Glu
rs146177313	39727699	c.3378C>A	Asn1126Lys
rs138611862	39727706	c.3385G>C	Asp1129His
rs150680317	39727758	c.3392G>A	Arg1131Gln
rs201482456	39727760	c.3394C>T	Pro1132Ser
rs150165942	39727538	c.3403C>T	Pro1135Ser
rs201374810	39727308	c.3416A>C	Asp1139Ala
rs1058808	39727784	c.3418C>G	Pro1140Ala
rs146177313	39727699	c.3423C>A	Asn1141Lys
rs141142822	39727317	c.3425T>C	Leu1142Pro
rs138611862	39727706	c.3430G>C	Asp1144His
rs376450229	39727325	c.3433G>A	Glu1145Lys
rs150680317	39727758	c.3437G>A	Arg1146Gln
rs201482456	39727760	c.3439C>T	Pro1147Ser
rs202149348	39727353	c.3461C>G	Ser1154Cys
rs1058808	39727784	c.3463C>G	Pro1155Ala
rs150680317	39727758	c.3482G>A	Arg1161Gln
rs201482456	39727760	c.3484C>T	Pro1162Ser
rs200796676	39727385	c.3493G>T	Asp1165Tyr
rs1058808	39727784	c.3508C>G	Pro1170Ala
rs373605104	39727887	c.3521C>G	Ala1174Gly
rs201353217	39727889	c.3523C>T	Pro1175Ser
rs373724781	39727423	c.3531G>C	Lys1177Asn
rs145772320	39727896	c.3530C>T	Pro1177Leu
rs200455249	39727440	c.3548C>T	Pro1183Leu
rs55943169	39727923	c.3557C>A	Ala1186Asp
rs111611886	39727448	c.3556G>A	Asp1186Asn
rs373605104	39727887	c.3566C>G	Ala1189Gly
rs201353217	39727889	c.3568C>T	Pro1190Ser
rs143958183	39727466	c.3574C>T	Arg1192Trp
rs145772320	39727896	c.3575C>T	Pro1192Leu
rs372043866	39727965	c.3599G>A	Arg1200Gln
rs55943169	39727923	c.3602C>A	Ala1201Asp
rs373605104	39727887	c.3611C>G	Ala1204Gly
rs201353217	39727889	c.3613C>T	Pro1205Ser
rs148211805	39727510	c.3618T>A	Asp1206Glu
rs145772320	39727896	c.3620C>T	Pro1207Leu
rs141494080	39727998	c.3632C>G	Pro1211Arg
rs144533600	39728006	c.3640G>A	Glu1214Lys
rs372043866	39727965	c.3644G>A	Arg1215Gln
rs55943169	39727923	c.3647C>A	Ala1216Asp
rs375408471	39728013	c.3647C>T	Pro1216Leu
rs150165942	39727538	c.3646C>T	Pro1216Ser
rs146177313	39727699	c.3666C>A	Asn1222Lys
rs184203026	39728030	c.3664G>A	Asp1222Asn
rs36085723	39728033	c.3667G>A	Val1223Met
rs138611862	39727706	c.3673G>C	Asp1225His
rs141494080	39727998	c.3677C>G	Pro1226Arg
rs144533600	39728006	c.3685G>A	Glu1229Lys
rs372043866	39727965	c.3689G>A	Arg1230Gln
rs375408471	39728013	c.3692C>T	Pro1231Leu
rs184203026	39728030	c.3709G>A	Asp1237Asn
rs36085723	39728033	c.3712G>A	Val1238Met

rs141494080	39727998	c.3722C>G	Pro1241Arg
rs150680317	39727758	c.3725G>A	Arg1242Gln
rs201482456	39727760	c.3727C>T	Pro1243Ser
rs144533600	39728006	c.3730G>A	Glu1244Lys
rs375408471	39728013	c.3737C>T	Pro1246Leu
rs1058808	39727784	c.3751C>G	Pro1251Ala
rs184203026	39728030	c.3754G>A	Asp1252Asn
rs36085723	39728033	c.3757G>A	Val1253Met
rs373605104	39727887	c.3854C>G	Ala1285Gly

rs201353217	39727889	c.3856C>T	Pro1286Ser
rs145772320	39727896	c.3863C>T	Pro1288Leu
rs55943169	39727923	c.3890C>A	Ala1297Asp
rs372043866	39727965	c.3932G>A	Arg1311Gln
rs141494080	39727998	c.3965C>G	Pro1322Arg
rs144533600	39728006	c.3973G>A	Glu1325Lys
rs375408471	39728013	c.3980C>T	Pro1327Leu
rs184203026	39728030	c.3997G>A	Asp1333Asn
rs36085723	39728033	c.4000G>A	Val1334Met

Supplemental Table 3: ErbB3 Protein changing polymorphism according to NCBI SNP Viewer

VariantID	Genomic position (Chr12)	Transcript change	Protein change
rs34379766	56080359	c.59C>A	Ser20Tyr
rs56017157	56083757	c.89C>T	Pro30Leu
rs374953448	56083789	c.121G>A	Asp41Asn
rs142735651	56083871	c.203C>T	Thr68Met
rs143770796	56083885	c.217G>A	Asp73Asn
rs143770796	56083885	c.217G>T	Asp73Tyr
rs77228285	56085025	c.265G>A	Val89Met
rs200856864	56085046	c.286A>G	Thr96Ala
rs201479792	56085062	c.302A>G	Asn101Ser
rs146486757	56085067	c.307C>T	Arg103Cys
rs984896	56085074	c.314T>G	Val105Gly
rs375644422	56085097	c.337G>C	Gly113Arg
rs199586304	56085224	c.464C>T	Ala155Val
rs368815108	56085227	c.467A>G	Gln156Arg
rs188955993	56085304	c.544G>A	Ala182Thr
rs374619740	56086546	c.437G>C	Gly146Ala
rs147905731	56086566	c.457G>A	Asp153Asn
rs141700623	56086578	c.469C>T	His157Tyr
rs188795493	56086590	c.481A>G	Ile161Val
rs374643843	56086605	c.496A>G	Ile166Val
rs200978269	56086618	c.509G>A	Arg170Gln
rs371016430	56086651	c.542G>A	Arg181Lys
rs368392747	56087577	c.548G>T	Cys183Phe
rs150454821	56087622	c.593G>T	Gly198Val
rs146860437	56087627	c.598G>A	Glu200Lys

VariantID	Genomic position (Chr12)	Transcript change	Protein change
rs386447328	56087640	c.611C>T	Thr204Ile
rs201079200	56087866	c.685G>A	Asp229Asn
rs376656358	56087872	c.691T>G	Cys231Gly
rs140656187	56087875	c.694G>T	Ala232Ser
rs374485725	56088028	c.740G>A	Arg247Gln
rs375740977	56088049	c.761C>A	Ala254Asp
rs370272057	56088075	c.787C>T	Leu263Phe
rs149635848	56088141	c.853G>A	Val285Ile
rs143406438	56088157	c.869G>A	Cys290Tyr
rs137870123	56088609	c.941A>G	Lys314Arg
rs372826813	56088625	c.957G>A	Met319Ile
rs375335792	56088638	c.970G>A	Gly324Arg
rs200211366	56088865	c.1106A>G	Asn369Ser
rs371094129	56092754	c.1117T>C	Trp373Arg
rs12320176	56092791	c.1154A>G	Asn385Ser
rs139868331	56092808	c.1171C>T	Arg391Trp
rs370847503	56093010	c.1208C>T	Pro403Leu
rs74763375	56093042	c.1240A>C	Asn414His
rs201880960	56093054	c.1252A>G	Ile418Val
rs141230043	56093055	c.1253T>A	Ile418Asn
rs375932235	56093346	c.1276C>T	Arg426Trp
rs144549266	56093428	c.1358G>A	Arg453His
rs200007116	56093436	c.1366A>G	Ile456Val
rs372171124	56093494	c.1424G>A	Arg475Gln
rs149951770	56093539	c.1469G>A	Arg490His

rs182692782	56093550	c.1480G>T	Val494Leu
rs375153329	56093772	c.1489G>A	Gly497Ser
rs146593760	56093776	c.1493A>T	Lys498Ile
rs145108143	56093821	c.1538G>A	Gly513Asp
rs200670489	56093874	c.1591A>T	Thr531Ser
rs147888915	56093880	c.1597T>C	Cys533Arg
rs267603578	56094124	c.1639G>A	Ala547Thr
rs201942735	56094137	c.1652C>T	Ser551Phe
rs370634710	56094146	c.1661C>T	Pro554Leu
rs202048840	56094166	c.1681G>A	Gly561Ser
rs368322587	56094402	c.1705G>C	Gly569Arg
rs370896872	56094420	c.1723C>G	Gln575Glu
rs141636701	56094426	c.1729G>A	Ala577Thr
rs371577741	56094435	c.1738C>T	Arg580Ter
rs200350558	56094436	c.1739G>A	Arg580Gln
rs373609369	56094528	c.1831C>T	Arg611Trp
rs200574817	56094537	c.1840C>G	His614Asp
rs143726790	56094540	c.1843G>A	Glu615Lys
rs151083303	56095268	c.1871C>G	Pro624Arg
rs141054346	56095300	c.1903G>A	Val635Met
rs371005132	56095676	c.1925T>C	Leu642Pro
rs369758674	56095730	c.1979G>C	Gly660Ala
rs139022684	56095732	c.1981G>A	Gly661Ser
rs200724560	56095756	c.2005C>T	Arg669Cys
rs373838865	56095773	c.2022A>C	Lys674Asn
rs56387488	56095798	c.2047C>T	Arg683Trp
rs138548737	56096505	c.2058C>G	Ser686Arg
rs181659329	56096522	c.2075C>A	Pro692His
rs368508813	56096539	c.2092A>G	Lys698Glu
rs371866354	56096563	c.2116G>C	Glu706Gln
rs35961836	56096597	c.2150C>T	Ser717Leu
rs189789018	56096614	c.2167G>C	Val723Leu
rs55787439	56096803	c.2231T>C	Ile744Thr
rs373831196	56096820	c.2248C>T	Arg750Trp
rs3891921	56096844	c.2272G>C	Asp758His
rs371109792	56097078	c.2308C>T	His770Tyr
rs202221237	56097109	c.2339G>A	Gly780Glu
rs144510847	56097153	c.2383C>G	Leu795Val
rs148448153	56097174	c.2404C>T	His802Tyr
rs375732870	56097176	c.2406C>G	His802Gln
rs182154425	56097181	c.2411G>T	Gly804Val
rs80185484	56097183	c.2413G>C	Ala805Pro
rs147206496	56097857	c.2533C>G	Pro845Ala
rs143021252	56097930	c.2606G>A	Ser869Asn
rs371250480	56098783	c.2717C>G	Thr906Ser
rs144558290	56098803	c.2737G>A	Ala913Thr
rs193920754	56098868	c.2802G>C	Gln934His
rs60586767	56099692	c.2884G>A	Ala962Thr
rs376939275	56099729	c.2921G>A	Arg974Gln
rs372311528	56099871	c.2971G>A	Glu991Lys
rs56259600	56099893	c.2993A>G	Lys998Arg
rs368317187	56099948	c.3048A>T	Glu1016Asp
rs139267530	56099957	c.3057G>C	Glu1019Asp
rs150001629	56099971	c.3071C>A	Thr1024Asn

rs370221639	56099988	c.3088G>A	Ala1030Thr
rs370849499	56100003	c.3103G>T	Val1035Phe
rs200017094	56100018	c.3118C>T	Arg1040Trp
rs149181380	56100019	c.3119G>A	Arg1040Gln
rs151311358	56100189	c.3145A>G	Ser1049Gly
rs373276051	56100202	c.3158G>C	Gly1053Ala
rs386542510	56100209	c.3165G>T	Met1055Ile
rs375145270	56100223	c.3179G>A	Gly1060Asp
rs112651994	56101088	c.3229C>T	Arg1077Trp
rs373840207	56101097	c.3238C>T	Arg1080Cys
rs376234145	56101098	c.3239G>A	Arg1080His
rs77822103	56101148	c.3289T>C	Ser1097Pro
rs138517750	56101211	c.3352C>T	Arg1118Trp
rs201958747	56101212	c.3353G>A	Arg1118Gln
rs773123	56101214	c.3355A>T	Ser1119Cys
rs201486425	56101236	c.3377C>T	Pro1126Leu
rs150312718	56101250	c.3391G>A	Ala1131Thr
rs79759315	56101265	c.3406C>T	Arg1136Cys
rs79759315	56101265	c.3406C>A	Arg1136Ser
rs149495975	56101308	c.3449G>C	Gly1150Ala
rs370634525	56101349	c.3490A>G	Thr1164Ala
rs180986542	56101543	c.3517C>T	Arg1173Trp
rs138861245	56101544	c.3518G>A	Arg1173Gln
rs375222170	56101553	c.3527C>A	Thr1176Asn
rs55699040	56101555	c.3529C>A	Leu1177Ile
rs147436223	56101613	c.3587A>G	Glu1196Gly
rs373454755	56101631	c.3605G>A	Arg1202Gln
rs371152673	56101777	c.3751A>C	Thr1251Pro
rs55709407	56101787	c.3761C>A	Thr1254Lys
rs377380727	56101805	c.3779A>T	Tyr1260Phe
rs267603579	56101815	c.3789G>A	Met1263Ile
rs369300309	56101819	c.3793C>T	Arg1265Trp
rs142809206	56101820	c.3794G>C	Arg1265Pro
rs11171743	56101837	c.3811G>A	Gly1271Ser
rs143742672	56101894	c.3868G>T	Glu1290Ter
rs146842909	56101939	c.3913G>A	Val1305Ile
rs376777418	56101984	c.3958G>C	Asp1320His
rs201199014	56102014	c.3988C>T	His1330Tyr
rs202205409	56102029	c.4003C>T	Pro1335Ser
rs140679673	56102051	c.4025C>T	Thr1342Met
rs144476801	56104806	c.69G>A	Met23Ile

Supplemental Table 4: ErbB4 Protein changing polymorphism according to NCBI SNP Viewer

VariantID	Genomic Position (Chr2)	Transcript change	Protein change
rs376416471	212538518	c.13A>G	Thr5Ala
rs201202926	212538481	c.50C>T	Ala17Val
rs373308672	212538473	c.58G>T	Val20Phe
rs2229091	212538468	c.63G>T	Gln21His
rs372631205	212124838	c.148C>T	Arg50Cys
rs368312588	212124773	c.213C>G	Asn71Lys
rs143662416	211947592	c.259G>T	Val87Leu
rs201678258	211947583	c.268G>T	Ala90Ser
rs201152419	211947544	c.307C>T	Arg103Cys
rs150757966	211947469	c.382A>G	Asn128Asp
rs192066345	211947459	c.392T>C	Leu131Pro
rs375361752	211788109	c.472G>A	Ala158Thr
rs370840578	211788108	c.473C>A	Ala158Glu
rs138433638	211788079	c.502C>T	Arg168Trp
rs200302763	211788078	c.503G>A	Arg168Gln
rs375307182	211788067	c.514C>T	Pro172Ser
rs78887537	211788043	c.538A>G	Thr180Ala
rs191386517	211750698	c.563G>A	Arg188His
rs369165560	211750674	c.587G>A	Arg196His
rs148992844	211725173	c.644A>C	Glu215Ala
rs376200174	211725087	c.730A>T	Thr244Ser
rs149800322	211722479	c.797T>G	Phe266Cys
rs201872526	211722450	c.826G>T	Glu276Ter
rs74898139	211722393	c.883C>T	His295Tyr
rs267599193	211713583	c.949G>A	Glu317Lys
rs148183215	211712152	c.1022C>T	Ser341Leu
rs374243191	211712104	c.1070T>C	Ile357Thr
rs76603692	211712052	c.1122T>G	His374Gln
rs138476466	211705366	c.1150A>G	Ile384Val
rs368876919	211705329	c.1187G>A	Arg396Lys
rs370646276	211702138	c.1318C>G	Gln440Glu
rs202247795	211702102	c.1354G>A	Glu452Lys
rs200092686	211702099	c.1357A>T	Ile453Phe
rs200755699	211702061	c.1395C>A	Asn465Lys
rs201156609	211702050	c.1406A>G	Tyr469Cys
rs149168827	211702042	c.1414A>G	Ile472Val
rs368860175	211702015	c.1441A>G	Ile481Val
rs376154931	211679148	c.1526G>A	Ser509Asn
rs371593463	211679103	c.1571G>A	Arg524His
rs141594820	211673253	c.1627T>A	Phe543Ile
rs267599192	211673250	c.1630C>T	Arg544Trp
rs200112693	211673218	c.1662G>C	Glu554Asp
rs376669939	211673212	c.1668C>A	Asp556Glu
rs374852421	211673200	c.1680G>C	Glu560Asp
rs146197533	211673193	c.1687G>A	Glu563Lys
rs147639134	211673171	c.1709A>G	His570Arg
rs200792124	211665474	c.1720C>A	Pro574Thr
rs373306524	211665414	c.1780C>A	Pro594Thr
rs199647352	211665368	c.1826A>G	Asp609Gly
rs149498255	211665363	c.1831G>A	Asp611Asn

rs139122290	211665359	c.1835G>A	Arg612Gln
rs190654033	211630569	c.1942A>T	Ile648Phe
rs190654033	211630569	c.1972A>T	Ile658Phe
rs190654033	211630569	c.2017A>T	Ile673Phe
rs145978648	211630566	c.1945G>T	Gly649Cys
rs145978648	211630566	c.1975G>T	Gly659Cys
rs145978648	211630566	c.2020G>T	Gly674Cys
rs370786638	211630545	c.1966A>G	Ile656Val
rs370786638	211630545	c.1996A>G	Ile666Val
rs370786638	211630545	c.2041A>G	Ile681Val
rs138313493	211630541	c.1970T>G	Val657Gly
rs138313493	211630541	c.2000T>G	Val667Gly
rs138313493	211630541	c.2045T>G	Val682Gly
rs202135721	211630507	c.2004G>C	Lys668Asn
rs202135721	211630507	c.2034G>C	Lys678Asn
rs202135721	211630507	c.2079G>C	Lys693Asn
rs150906199	211630472	c.2039T>G	Leu680Trp
rs150906199	211630472	c.2069T>G	Leu690Trp
rs150906199	211630472	c.2114T>G	Leu705Trp
rs142249882	211624026	c.2068C>T	Pro690Ser
rs142249882	211624026	c.2098C>T	Pro700Ser
rs142249882	211624026	c.2143C>T	Pro715Ser
rs267599191	211623993	c.2101C>T	Arg701Cys
rs267599191	211623993	c.2131C>T	Arg711Cys
rs267599191	211623993	c.2176C>T	Arg726Cys
rs138873985	211623960	c.2134A>G	Lys712Glu
rs138873985	211623960	c.2164A>G	Lys722Glu
rs138873985	211623960	c.2209A>G	Lys737Glu
rs374970657	211623932	c.2162C>T	Thr721Met
rs374970657	211623932	c.2192C>T	Thr731Met
rs374970657	211623932	c.2237C>T	Thr746Met
rs141502430	211619230	c.2218A>G	Ile740Val
rs141502430	211619230	c.2248A>G	Ile750Val
rs141502430	211619230	c.2293A>G	Ile765Val
rs182073178	211619206	c.2242G>T	Gly748Cys
rs182073178	211619206	c.2272G>T	Gly758Cys
rs182073178	211619206	c.2317G>T	Gly773Cys
rs375308478	211619191	c.2257G>A	Val753Met
rs375308478	211619191	c.2287G>A	Val763Met
rs375308478	211619191	c.2332G>A	Val778Met
rs201419322	211562073	c.2287G>T	Ala763Ser
rs201419322	211562073	c.2317G>T	Ala773Ser
rs201419322	211562073	c.2317G>A	Ala773Thr
rs201419322	211562073	c.2362G>T	Ala788Ser
rs201419322	211562073	c.2362G>A	Ala788Thr
rs367778613	211561994	c.2366T>C	Met789Thr
rs367778613	211561994	c.2396T>C	Met799Thr
rs367778613	211561994	c.2441T>C	Met814Thr
rs112196222	211561949	c.2411A>G	Asn804Ser
rs112196222	211561949	c.2486A>G	Asn829Ser
rs112196222	211561949	c.2441A>G	Asn814Ser
rs373311688	211561911	c.2449A>G	Ile817Val
rs373311688	211561911	c.2479A>G	Ile827Val
rs373311688	211561911	c.2524A>G	Ile842Val

rs369248674	211431070	c.2488G>A	Val830Ile
rs369248674	211431070	c.2518G>A	Val840Ile
rs369248674	211431070	c.2563G>A	Val855Ile
rs181319801	211428455	c.2672G>A	Cys891Tyr
rs181319801	211428455	c.2717G>A	Cys906Tyr
rs181319801	211428455	c.2642G>A	Cys881Tyr
rs149713914	211428453	c.2644A>G	Ile882Val
rs149713914	211428453	c.2674A>G	Ile892Val
rs149713914	211428453	c.2719A>G	Ile907Val
rs368984080	211428414	c.2683A>G	Ser895Gly
rs368984080	211428414	c.2713A>G	Ser905Gly
rs368984080	211428414	c.2758A>G	Ser920Gly
rs374581758	211424279	c.2712G>T	Met904Ile
rs374581758	211424279	c.2742G>T	Met914Ile
rs374581758	211424279	c.2787G>T	Met929Ile
rs148791072	211424244	c.2747C>T	Thr916Met
rs148791072	211424244	c.2777C>T	Thr926Met
rs148791072	211424244	c.2822C>T	Thr941Met
rs397514262	211424241	c.2750G>A	Arg917Gln
rs397514262	211424241	c.2780G>A	Arg927Gln
rs397514262	211424241	c.2825G>A	Arg942Gln
rs147624110	211424215	c.2776G>A	Gly926Arg
rs147624110	211424215	c.2806G>A	Gly936Arg
rs147624110	211424215	c.2851G>A	Gly951Arg
rs370965857	211424181	c.2810T>C	Ile937Thr
rs370965857	211424181	c.2840T>C	Ile947Thr
rs370965857	211424181	c.2885T>C	Ile962Thr

rs376298364	211424176	c.2815G>A	Val939Ile
rs376298364	211424176	c.2845G>A	Val949Ile
rs376298364	211424176	c.2890G>A	Val964Ile
rs143134749	211420602	c.3019C>T	Arg1007Cys
rs143134749	211420602	c.2944C>T	Arg982Cys
rs143134749	211420602	c.2974C>T	Arg992Cys
rs112008136	211420553	c.3023T>G	Leu1008Trp
rs112008136	211420553	c.3068T>G	Leu1023Trp
rs112008136	211420553	c.2993T>G	Leu998Trp
rs139001004	211420526	c.3020T>C	Met1007Thr
rs139001004	211420526	c.3050T>C	Met1017Thr
rs139001004	211420526	c.3095T>C	Met1032Thr
rs369858826	211420484	c.3062C>G	Pro1021Arg
rs369858826	211420484	c.3092C>G	Pro1031Arg
rs369858826	211420484	c.3137C>G	Pro1046Arg
rs377159914	211420472	c.3074A>C	Tyr1025Ser
rs377159914	211420472	c.3104A>C	Tyr1035Ser
rs377159914	211420472	c.3149A>C	Tyr1050Ser
rs146491343	211420449	c.3097T>C	Ser1033Pro
rs146491343	211420449	c.3127T>C	Ser1043Pro
rs146491343	211420449	c.3172T>C	Ser1058Pro
rs202053701	211420442	c.3104G>A	Arg1035Lys
rs202053701	211420442	c.3104G>C	Arg1035Thr
rs202053701	211420442	c.3134G>A	Arg1045Lys
rs202053701	211420442	c.3134G>C	Arg1045Thr
rs202053701	211420442	c.3179G>A	Arg1060Lys
rs202053701	211420442	c.3179G>C	Arg1060Thr

rs200172669	211387148	c.3108C>A	Asn1036Lys
rs200172669	211387148	c.3138C>A	Asn1046Lys
rs200172669	211387148	c.3156C>A	Asn1052Lys
rs200172669	211387148	c.3183C>A	Asn1061Lys
rs200172669	211387148	c.3186C>A	Asn1062Lys
rs200172669	211387148	c.3231C>A	Asn1077Lys
rs149665378	211387141	c.3115G>T	Val1039Leu
rs149665378	211387141	c.3145G>T	Val1049Leu
rs149665378	211387141	c.3163G>T	Val1055Leu
rs149665378	211387141	c.3190G>T	Val1064Leu
rs149665378	211387141	c.3193G>T	Val1065Leu
rs149665378	211387141	c.3238G>T	Val1080Leu
rs147514533	211387134	c.3122G>A	Arg1041Gln
rs147514533	211387134	c.3152G>A	Arg1051Gln
rs147514533	211387134	c.3170G>A	Arg1057Gln
rs147514533	211387134	c.3197G>A	Arg1066Gln
rs147514533	211387134	c.3200G>A	Arg1067Gln
rs147514533	211387134	c.3245G>A	Arg1082Gln
rs143251275	211387081	c.3175A>G	Thr1059Ala
rs143251275	211387081	c.3205A>G	Thr1069Ala
rs143251275	211387081	c.3223A>G	Thr1075Ala
rs143251275	211387081	c.3250A>G	Thr1084Ala
rs143251275	211387081	c.3253A>G	Thr1085Ala
rs143251275	211387081	c.3298A>G	Thr1100Ala
rs182276587	211387077	c.3179G>A	Ser1060Asn
rs182276587	211387077	c.3209G>A	Ser1070Asn
rs182276587	211387077	c.3227G>A	Ser1076Asn
rs182276587	211387077	c.3254G>A	Ser1085Asn
rs182276587	211387077	c.3257G>A	Ser1086Asn
rs182276587	211387077	c.3302G>A	Ser1101Asn
rs372258619	211387059	c.3197C>A	Pro1066His
rs372258619	211387059	c.3227C>A	Pro1076His
rs372258619	211387059	c.3245C>A	Pro1082His
rs372258619	211387059	c.3272C>A	Pro1091His
rs372258619	211387059	c.3275C>A	Pro1092His
rs372258619	211387059	c.3320C>A	Pro1107His
rs144311212	211387000	c.3256C>T	Arg1086Cys
rs144311212	211387000	c.3286C>T	Arg1096Cys
rs144311212	211387000	c.3304C>T	Arg1102Cys
rs144311212	211387000	c.3331C>T	Arg1111Cys
rs144311212	211387000	c.3334C>T	Arg1112Cys
rs144311212	211387000	c.3379C>T	Arg1127Cys
rs139785964	211386934	c.3322G>A	Val1108Met
rs139785964	211386934	c.3352G>A	Val1118Met
rs139785964	211386934	c.3370G>A	Val1124Met
rs139785964	211386934	c.3397G>A	Val1133Met
rs139785964	211386934	c.3400G>A	Val1134Met
rs139785964	211386934	c.3445G>A	Val1149Met
rs78030748	211386924	c.3332C>T	Pro1111Leu
rs78030748	211386924	c.3362C>T	Pro1121Leu
rs78030748	211386924	c.3380C>T	Pro1127Leu
rs78030748	211386924	c.3407C>T	Pro1136Leu
rs78030748	211386924	c.3410C>T	Pro1137Leu
rs78030748	211386924	c.3455C>T	Pro1152Leu

rs368679346	211386919	c.3337C>T	Arg1113Trp
rs368679346	211386919	c.3367C>T	Arg1123Trp
rs368679346	211386919	c.3385C>T	Arg1129Trp
rs368679346	211386919	c.3412C>T	Arg1138Trp
rs368679346	211386919	c.3415C>T	Arg1139Trp
rs368679346	211386919	c.3460C>T	Arg1154Trp
rs377737065	211386918	c.3338G>A	Arg1113Gln
rs377737065	211386918	c.3368G>A	Arg1123Gln
rs377737065	211386918	c.3386G>A	Arg1129Gln
rs377737065	211386918	c.3413G>A	Arg1138Gln
rs377737065	211386918	c.3416G>A	Arg1139Gln
rs377737065	211386918	c.3461G>A	Arg1154Gln
rs3748961	211386909	c.3347G>A	Arg1116Gln
rs3748961	211386909	c.3377G>A	Arg1126Gln
rs3748961	211386909	c.3395G>A	Arg1132Gln
rs3748961	211386909	c.3422G>A	Arg1141Gln
rs3748961	211386909	c.3425G>A	Arg1142Gln
rs3748961	211386909	c.3470G>A	Arg1157Gln
rs140366015	211386883	c.3373A>G	Met1125Val
rs140366015	211386883	c.3403A>G	Met1135Val
rs140366015	211386883	c.3421A>G	Met1141Val
rs140366015	211386883	c.3448A>G	Met1150Val
rs140366015	211386883	c.3451A>G	Met1151Val
rs140366015	211386883	c.3496A>G	Met1166Val
rs373818748	211386867	c.3389A>G	Asp1130Gly
rs373818748	211386867	c.3419A>G	Asp1140Gly
rs373818748	211386867	c.3437A>G	Asp1146Gly
rs373818748	211386867	c.3464A>G	Asp1155Gly
rs373818748	211386867	c.3467A>G	Asp1156Gly
rs373818748	211386867	c.3512A>G	Asp1171Gly
rs202042335	211384048	c.3416C>G	Pro1139Arg
rs202042335	211384048	c.3446C>G	Pro1149Arg
rs202042335	211384048	c.3464C>G	Pro1155Arg
rs202042335	211384048	c.3491C>G	Pro1164Arg
rs202042335	211384048	c.3494C>G	Pro1165Arg
rs202042335	211384048	c.3539C>G	Pro1180Arg
rs144842611	211383983	c.3481G>C	Glu1161Gln
rs144842611	211383983	c.3511G>C	Glu1171Gln
rs144842611	211383983	c.3529G>C	Glu1177Gln
rs144842611	211383983	c.3556G>C	Glu1186Gln
rs144842611	211383983	c.3559G>C	Glu1187Gln
rs144842611	211383983	c.3604G>C	Glu1202Gln
rs7425801	211383982	c.3482A>G	Glu1161Gly
rs7425801	211383982	c.3512A>G	Glu1171Gly
rs7425801	211383982	c.3530A>G	Glu1177Gly
rs7425801	211383982	c.3557A>G	Glu1186Gly
rs7425801	211383982	c.3560A>G	Glu1187Gly
rs7425801	211383982	c.3605A>G	Glu1202Gly
rs201275165	211383964	c.3500A>G	Asn1167Ser
rs201275165	211383964	c.3530A>G	Asn1177Ser
rs201275165	211383964	c.3548A>G	Asn1183Ser
rs201275165	211383964	c.3575A>G	Asn1192Ser
rs201275165	211383964	c.3578A>G	Asn1193Ser
rs201275165	211383964	c.3623A>G	Asn1208Ser

rs150628313	211383796	c.3668C>T	Pro1223Leu
rs150628313	211383796	c.3698C>T	Pro1233Leu
rs150628313	211383796	c.3716C>T	Pro1239Leu
rs150628313	211383796	c.3743C>T	Pro1248Leu
rs150628313	211383796	c.3746C>T	Pro1249Leu
rs150628313	211383796	c.3791C>T	Pro1264Leu
rs371332509	211383728	c.3736G>A	Gly1246Arg
rs371332509	211383728	c.3766G>A	Gly1256Arg
rs371332509	211383728	c.3784G>A	Gly1262Arg
rs371332509	211383728	c.3811G>A	Gly1271Arg
rs371332509	211383728	c.3814G>A	Gly1272Arg
rs371332509	211383728	c.3859G>A	Gly1287Arg
rs397514263	211383719	c.3745C>T	Arg1249Trp
rs397514263	211383719	c.3775C>T	Arg1259Trp
rs397514263	211383719	c.3793C>T	Arg1265Trp
rs397514263	211383719	c.3820C>T	Arg1274Trp
rs397514263	211383719	c.3823C>T	Arg1275Trp
rs397514263	211383719	c.3868C>T	Arg1290Trp
rs141902440	211383718	c.3746G>A	Arg1249Gln
rs141902440	211383718	c.3776G>A	Arg1259Gln
rs141902440	211383718	c.3794G>A	Arg1265Gln
rs141902440	211383718	c.3821G>A	Arg1274Gln
rs141902440	211383718	c.3824G>A	Arg1275Gln
rs141902440	211383718	c.3869G>A	Arg1290Gln
rs367568427	211383683	c.3781G>A	Glu1261Lys
rs367568427	211383683	c.3811G>A	Glu1271Lys
rs367568427	211383683	c.3829G>A	Glu1277Lys
rs367568427	211383683	c.3856G>A	Glu1286Lys
rs367568427	211383683	c.3859G>A	Glu1287Lys
rs367568427	211383683	c.3904G>A	Glu1302Lys

1
